# Diseased Erythrocyte Enrichment Based on I-Shaped Pillar DLD Arrays

**DOI:** 10.3390/mi15020214

**Published:** 2024-01-31

**Authors:** Yao Lv, Jiangbo Wu, Yongqing He, Jie Liu, Wenyu Zhang, Zihan Yan

**Affiliations:** 1 School of Energy and Power Engineering, Lanzhou University of Technology, Lanzhou 730050, China; ly@lut.edu.cn (Y.L.); ahriempress1314@gmail.com (J.L.); zwy@lut.edu.cn (W.Z.); wzyzh5246@gmail.com (Z.Y.); 2 Chongqing Key Laboratory of Micro-Nano System and Intelligent Sensing, Chongqing Technology and Business University, Chongqing 400067, China

**Keywords:** deterministic lateral displacement, finite element cell model, erythrocyte deformability

## Abstract

Enrichment of erythrocytes is a necessary step in the diagnosis of blood diseases. Due to the high deformability and viscosity of erythrocytes, they cannot be regarded as stable point-like solids, so the influence of their deformability on fluid dynamics must be considered. Therefore, by using the special effect of an I-shaped pillar (I-pillar) on erythrocytes, erythrocytes with different deformability can be made to produce different provisional distances in the chip, so as to achieve the separation of the two kinds of erythrocytes. In this study, a microfluidic chip was designed to conduct a control test between erythrocytes stored for a long time and fresh erythrocytes. At a specific flow rate, the different deformable erythrocytes in the chip move in different paths. Then, the influence of erythrocyte deformability on its movement trajectory was analyzed by two-dimensional finite element flow simulation. DLD sorting technology provides a new method for the sorting and enrichment of diseased erythrocytes.

## 1. Introduction

Erythrocytes are an important part of human blood, playing a crucial role as the primary transporters of oxygen and carbon dioxide [1,2,3]. Erythrocyte transfusion is the focus of modern clinical blood transfusion, representing the primary element in component blood transfusion, and is extensively studied and applied [4,5,6]. The deformability of erythrocytes can be significantly impaired by a variety of diseases [7,8,9], and the deformability of erythrocytes significantly affects blood rheology. Additionally, following blood collection and storage in the blood bank, hemorheological properties undergo changes over time: whole blood viscosity, plasma viscosity, erythrocyte deformability, and other parameters gradually increase [10,11,12]. Various blood components lose regulation by the nervous system and body fluids after isolation, disrupting the biochemical activity of blood cells. These factors impact the quality of blood transfusion.

Recent reports highlight the potential harm of allogeneic erythrocyte transfusion to blood recipients. Reilly M. et al. [13] summarized erythrocyte storage lesions and related clinical data, and found that changes in erythrocyte storage can reduce transfusion efficiency and even induce adverse events. The reasons are complex and varied. Consequently, there is a need to investigate the rheological properties of hardened erythrocytes.

As a new technology in recent years, the microfluidic method can potentially apply tiny biological particles. The application of microfluidic technology can provide a simple and efficient new method for the research of blood diseases compared to the complicated operations and expensive cell detection instruments that are widely used nowadays. Yang Y.P. et al. [14] combined microfluidics and deep learning algorithms to recognize and classify erythrocytes based on morphology. This was used to rapidly evaluate the quality of stored erythrocyte samples. The developed target detection model achieves an average accuracy of 89.24%. Among these technologies, a microfluidic technique called deterministic lateral displacement (DLD) is capable of separating and enriching micro-nano particles of varying sizes [15]. By arranging arrays of micropillars arranged in a homing arrangement in the chip, the DLD microfluidic chip can control the flow of particles of a specific size in a suspension to a specific outlet.

Since the inception of DLD technology, numerous advancements in pillar designs and array layouts have made the technique applicable to non-spherical particles, such as erythrocytes. Davis J.A. et al. [16] separated leukocytes, erythrocytes, and platelets from virtually undiluted blood using a DLD array constructed from cylinders at a processing rate of 1μL/min. The wing-and-rhomboid pillar shapes introduced by Al-Fandi et al. [17] were compared with circular pillars, and the results showed that winged pillars can reduce the deformation of biological particles during flow, mentioning the ability of the chip to separate blood cells from trypanosomes. Zeming K.K. et al. [18] demonstrated that an I-shaped pillar structure induces erythrocytes to flow continuously to the side of the main channel in a displacement mode. Its maximum size was utilized by inducing erythrocytes to roll near the pillar.

In a DLD chip, the deformation of cells under stress is affected by many factors. In order to more deeply explore the influence of fluid change in DLD chips on erythrocyte motion path, more numerical methods have also been used to study fluid flow in DLD chips, among which many flow simulation designs using the finite element method have been born. Kabacaoglu G. et al. [19] showed that soft cells and rigid cells undergo different deformations when passing through a gap, which causes the two types of cells to tend to flow in different directions. Zhang Z. et al. [20] found that the high deformability of erythrocytes allows them to change flow direction under the action of fluids.

If the characteristics of the I-pillar can be used to induce erythrocytes to fold and deform near the pillar, then these cells can obtain different provisional distances. By designing the corresponding DLD array structure with these provisional distances, the sorting of different deformable erythrocytes can be realized. Therefore, we developed a DLD chip that uses I-pillars to sort erythrocytes of different deformability. The relationship between the motion path of different deformable erythrocyte models and the flow field inside the chip was analyzed by two-dimensional finite element simulation.

## 2. Layout Design of the Array in the Chip

By comprehensively analyzing the current experimental process involving erythrocytes and microfluidic chips, this paper serves as the foundation for experimental research. The design of the experimental system commences with the selection of the chip production method and systematically progresses through the construction of the experimental setup.

### 2.1. Size Design of Sorting Chip

**Figure 1:**
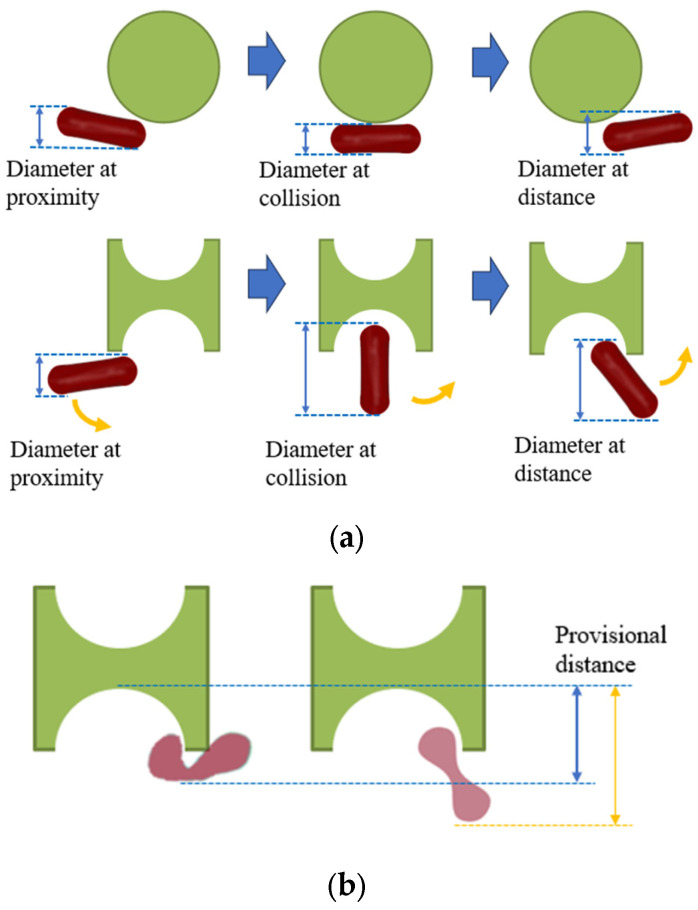
Effect of I-pillar on erythrocyte effective diameter. (**a**) The magnification effect of I-pillar on effective DLD diameter of erythrocytes was demonstrated; (**b**) Erythrocytes of different deformability acquired different effective diameters on collision with the pillar.

As depicted in Figure 1, the distinctive shape of the I-shaped pillar induces tumbling of erythrocytes during flow, contrasting with the conventional pillar shape. This allows for more efficient utilization of the maximum size of this non-spherical particle. If the tumbling motion, as briefly illustrated in Figure 1b, can be applied at the groove of the I-pillar to generate varying folding deformations in erythrocytes with different deformabilities, then the two kinds of erythrocytes can obtain different paths through different provisional distances.

**Figure 2:**
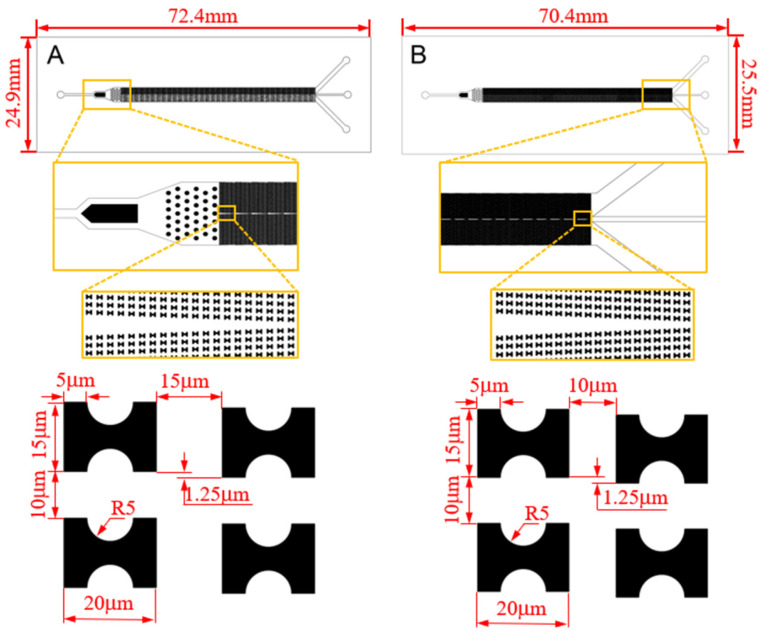
CAD structure drawing and size parameters of the chips.

With this effect in mind, we carry out the design of the sorting chip. Among various microfluidic chip fabrication methods, the PDMS microfluidic chip produced through soft lithography technology is chosen due to its advantageous features such as robust plasticity, elasticity, and cell-friendly properties [21,22,23]. This method will be employed for the chip fabrication. Drawing on our existing flow simulation experience, we have devised two chips, labeled as A and B, depicted in Figure 2.

### 2.2. Fabrication of Sorting Chip

**Figure 3:**
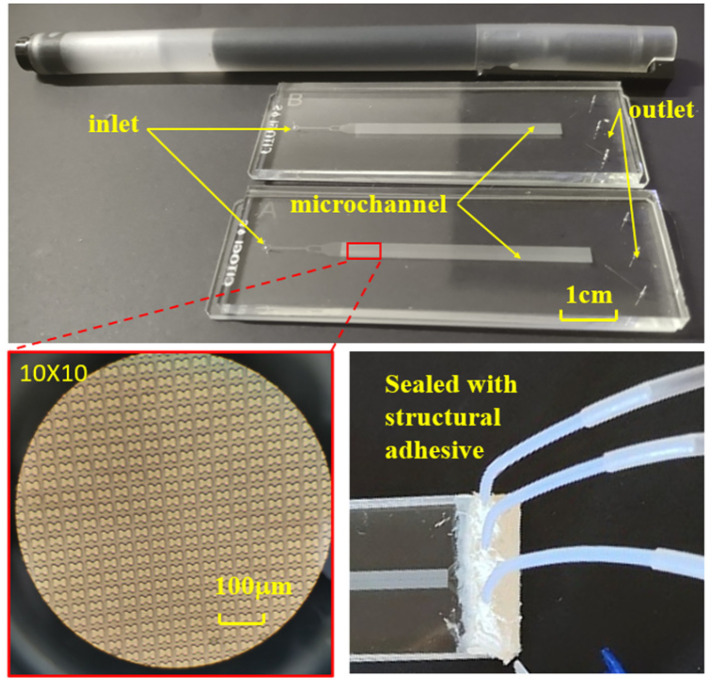
Finished chip, internal microstructure, and pipe connection.

The chip’s internal structure comprises an initial inlet, a rectifier pillar array in the front part of the channel, a DLD array in the middle of the channel, and three outlets at the end. To enhance the dispersion of particles in the suspension and ensure uniform distribution of erythrocyte samples at the array inlet, a rectification structure is strategically positioned between the erythrocyte suspension chip inlet and the array. Furthermore, a mirror structure is employed during the array arrangement. The upper section of the mirrored array guides erythrocytes downward towards the central wall, while the lower section directs them upward. This design effectively doubles the device width without increasing the array length, a critical consideration for ensuring the displacement of all target cells to the central region, typically achieved by scaling the array width divided by the array tilt. The process of fabricating a microfluidic chip through soft lithography comprises three main stages: creating a template using photoresist, using PDMS material to replicate the template and form the top layer of the chip, and finally, enclosing the fabricated top layer with a glass sheet to produce a complete chip, as depicted in Figure 3. To establish fluidic connections, holes are punched through the PDMS chip. Subsequently, the open channels are sealed with a peeling foil, as shown in Figure 3, using metal pins to connect the chip through the punch holes. Figure 3 illustrates the PDMS chip with its microchannels, easily accessible through the punch holes. The outer dimensions of the PDMS chip align with those of a standard cover slip, facilitating seamless integration into an experimental setup that utilizes a microscope for visualizing erythrocytes.

## 3. Erythrocyte Enrichment Experiment

The erythrocyte suspension is prepared after the fabrication of the chip. To investigate the impact of storage time on the rheological characteristics of erythrocytes, we conduct flow tests separately for erythrocytes stored for a specific duration and fresh erythrocytes. We compare the deformation and paths of the two erythrocyte types during flow. Subsequently, we assess the chip’s performance based on the enrichment effect observed with different erythrocytes.

### 3.1. Preparation of the Experiment

Erythrocyte samples were obtained from fresh venous blood of volunteers. The venous blood underwent centrifugation to produce an erythrocyte suspension with isotonic PBS buffer, which was then refrigerated at 4 °C [10,24,25]. To make hardened erythrocytes, erythrocyte samples stored for 1 day (1D) and 7 days (7D) were selected for control tests [26,27]. A 1 mL erythrocyte suspension containing 10^7^ cells was diluted to 10 mL with PBS buffer.

The syringe pump injected the suspension at a flow rate set to 4.5 μL/min, and the inlet mean fluid flow rate *u*_0_ was calculated to be approximately 1.5 mm/s based on the cross-sectional area of the microchannel. When the system reached a stable working condition, erythrocyte distribution was captured at the central flow channel at the entrance, middle, and end of the array, as well as at the boundary flow channel every 10 s. Three sets of data were recorded to statistically analyze the array’s erythrocyte enrichment efficiency. To mitigate the impact of the mirror structure’s center on the flow, the image was captured at the selected center position on both sides of the array. Data were collected from the front, middle, and back of the array, with image data gathered three times in each group. The injection pump flow rate was adjusted to 3 μL/min and 1.5 μL/min (about *u*_0_ = 1 mm/s and *u*_0_ = 0.5 mm/s) for the erythrocyte suspension, and the enrichment efficiency and erythrocyte motion path were recorded at each flow rate. Following these steps, another chip or erythrocytes with different storage times were introduced, and changes in erythrocyte motion path were documented under varying parameters.

### 3.2. Experimental Results

**Figure 4:**
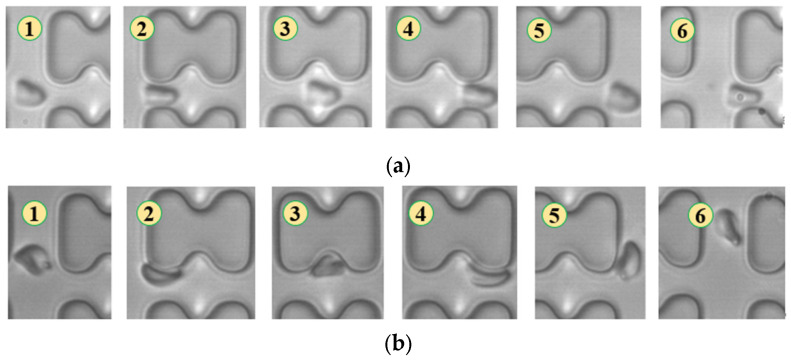
Flow of two types of erythrocytes in the A-chip when the injection rate was 4.5 μL/min, the numbers in the figure show the change in shape and position of the erythrocytes as they pass near a pillar. (**a**) 1D erythrocytes; (**b**) 7D erythrocytes.

**Figure 5:**
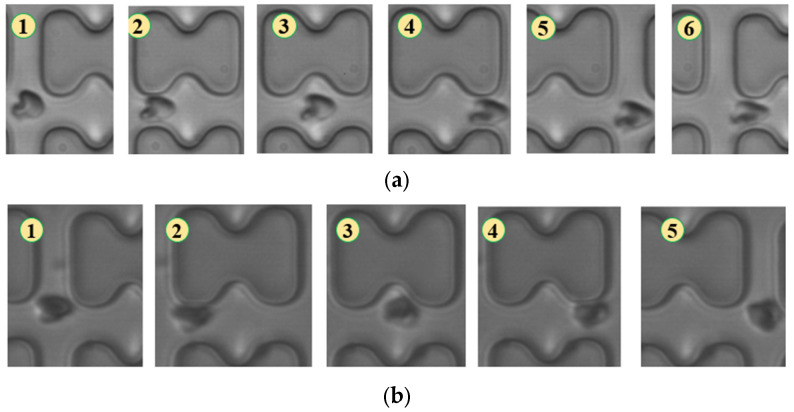
Flow of two types of erythrocytes in the B-chip when the injection rate was 4.5 μL/min, the numbers in the figure show the change in shape and position of the erythrocytes as they pass near a pillar. (**a**) 1D erythrocytes; (**b**) 7D erythrocytes.

**Figure 6:**
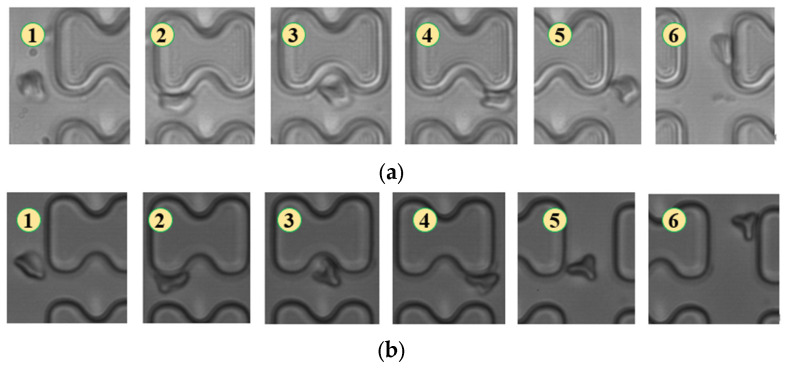
Flow of two types of erythrocytes in the A-chip when the injection rate was 3 μL/min, the numbers in the figure show the change in shape and position of the erythrocytes as they pass near a pillar. (**a**) 1D erythrocytes; (**b**) 7D erythrocytes.

**Figure 7:**
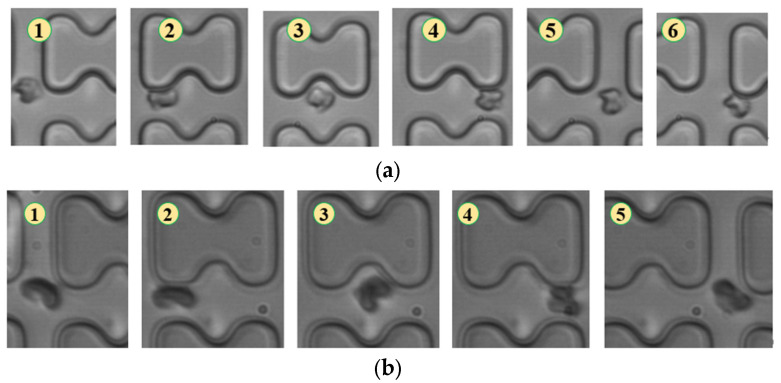
Flow of two types of erythrocytes in the B-chip when the injection rate was 3 μL/min, the numbers in the figure show the change in shape and position of the erythrocytes as they pass near a pillar. (**a**) 1D erythrocytes; (**b**) 7D erythrocytes.

**Figure 8:**
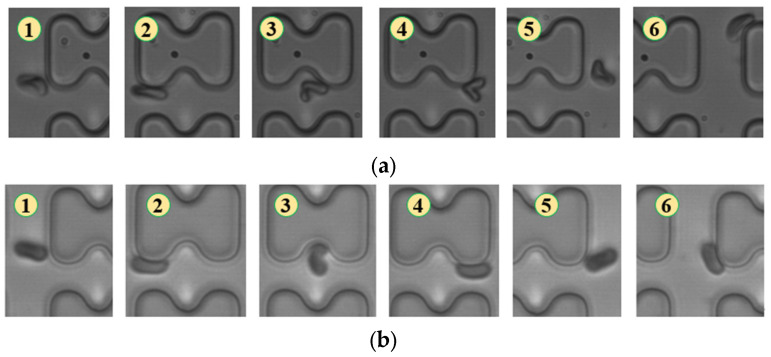
Flow of two types of erythrocytes in the A-chip when the injection rate was 1.5 μL/min, the numbers in the figure show the change in shape and position of the erythrocytes as they pass near a pillar. (**a**) 1D erythrocytes; (**b**) 7D erythrocytes.

**Figure 9:**
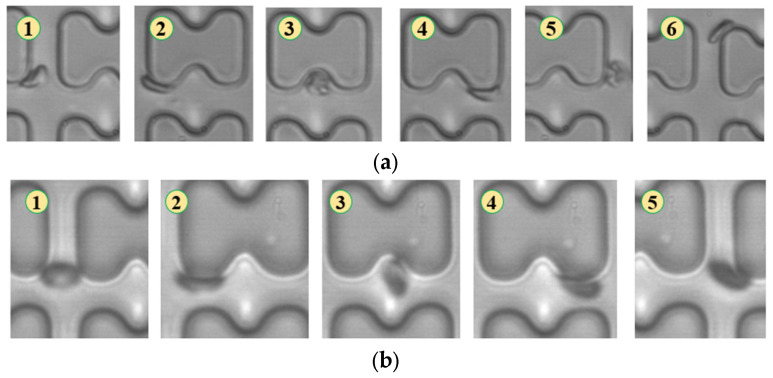
Flow of two types of erythrocytes in the B-chip when the injection rate was 1.5 μL/min, the numbers in the figure show the change in shape and position of the erythrocytes as they pass near a pillar. (**a**) 1D erythrocytes; (**b**) 7D erythrocytes.

In Figure 4, chip A, featuring a large transverse gap in the array, exhibits a high-speed fluid impact on most 1D erythrocytes at the center of the longitudinal gap. This leads to an exaggerated “parachute”-like deformation of erythrocytes. Erythrocytes in this state occupy nearly the entire longitudinal gap of the array and continue to flow in a displacement mode, primarily influenced by the central high-speed fluid. The “parachute” type produced by the 7D erythrocytes is less varied. After colliding with the pillar, the erythrocytes undergo violent folding, assuming a “string ball” shape, and flow in a zigzag pattern near the pillar under the influence of the surrounding fluid. The numbers in Figure 4, Figure 5, Figure 6, Figure 7, Figure 8 and Figure 9 indicate the change in shape and position of the erythrocytes in the vicinity of a given pillar as time advances.

In Figure 5, the deformation of the three types of erythrocytes in chip B, which has a smaller transverse clearance, was similar to that in chip A, but all of them flowed in displacement mode.

When the injection rate was 3 μL/min, the deformation of all three types of erythrocytes decreased, with 1D erythrocytes deforming similarly to 7D erythrocytes, gradually adopting a “parachute”-like deformation through continued collisions with the pillar. The flow trajectories of the two types of erythrocytes showed no significant difference, as depicted in Figure 6.

As shown in Figure 7, in chip B, the reduced distance between rows prevented erythrocytes 1D and 7D from breaking away from the “string ball” shape, maintaining a chaotic folding state and flowing in displacement mode throughout the flow process.

With a further reduction in flow rate to 1.5 μL/min, the degree of deformation of both erythrocytes decreased. Both 1D and 7D erythrocytes maintained a pie-like shape, differing only in the amount of deformation quantity upon collision with the pillar, as shown in Figure 8.

In Figure 9, the flow of the two types of erythrocytes in the B-chip is depicted. The flow of 1D erythrocytes is similar to that in the A-chip, proceeding in a zigzag pattern by conforming to the pillars. The 7D erythrocytes flow in a displacement mode in a rolling posture.

**Figure 10:**
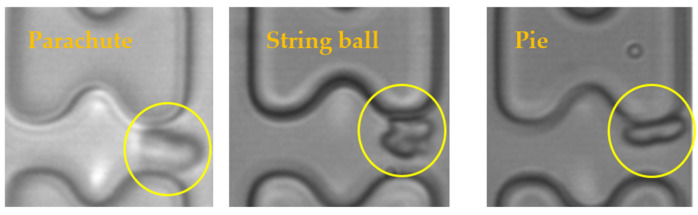
Three shapes of erythrocytes (inside yellow circle) in the chip.

The results of the experiments were analyzed, and it was found that the shapes of the erythrocytes in the chip could be roughly classified into three categories, as shown in Figure 10. The parachute-shaped erythrocytes in the figure were formed by being held up and elongated from the center by the high-speed water flow, and the erythrocytes in this state tend to follow the high-speed water flow in the middle of the flow channel. The string ball-shaped erythrocytes are formed in a more watery current by the constant pulling of the current as well as the collision of the pillars, and this state of the erythrocytes is characterized by a greater randomness in their trajectory through the array due to the rapid change in shape. When the flow rate in the chip is small, the erythrocytes can maintain a pie-shaped structure in the array, and the erythrocytes in this state will undergo a rolling motion under the action of the I-pillars.

The deformation of erythrocytes as well as the main trajectories in each group of experiments were organized, and the results are shown in Table 1.

**Figure 11:**
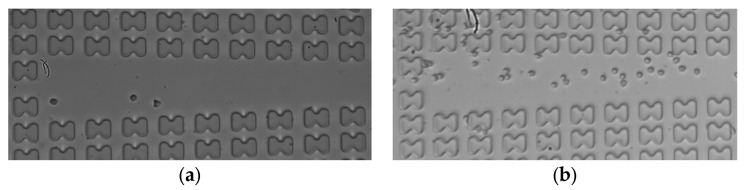
The 7D erythrocytes enrichment effect in the A-chip when the flow rate is 3 μL/min. (**a**) erythrocyte distribution at the entrance of the array; (**b**) erythrocyte distribution at the end of the array.

Subsequently, the erythrocyte enrichment effects of the two microchips were measured at various flow rates. Figure 11 illustrates the observation of the number of erythrocytes in the center channel of array mirror-symmetry for 7D erythrocytes in the A-chip at a flow rate of 3 μL/min. A comparison was made between the number of erythrocytes at the front and the end of the array within one cycle. The cell enrichment effects of the first and last three groups were calculated and averaged under each working condition.

The enrichment multiplicity of the two types of erythrocytes within both the A- and B-chip was counted and is presented in Table 2.

By comparing the experimental cell flow trajectories with the final enrichment efficiency, it can be observed that, due to differences in deformability, 1D erythrocytes in the A-chip flowed in a displacement mode under exaggerated “parachute” deformation at the inlet flow rate of 4.5 μL/min. This behavior differed from that of 7D erythrocytes, which flowed in a zigzag mode, primarily in the form of “string ball” under the same conditions. The enrichment multiplicity of 1D erythrocytes was more than three times that of 7D erythrocytes. The 1D erythrocytes in the A-chip flowed in a displacement mode under exaggerated “parachute” deformation, differing from the zigzag flow of 7D erythrocytes under the same conditions. The enrichment multiplicity of 7D erythrocytes in the A-chip was more than three times that of the latter.

At an inlet flow rate of 1.5 μL/min, 1D erythrocytes tended to flow in zigzag mode in both the A- and B-chip, while 7D erythrocytes tended to flow in displacement mode due to the difference in morphology during tumbling flow. The multiplicity of enrichment of 7D erythrocytes was about twice as high as that of 1D erythrocytes in the A-chip and about three times as high in the B-chip. Therefore, achieving the separation of erythrocytes with different deformability by I-pillar DLD arrays at higher or lower flow rates is feasible.

Comparing their effects, the two kinds of chips have different enrichment effects on different deformable erythrocytes under the corresponding flow rate parameters, which can achieve the distinction. However, the array gap in chip A means that it can only achieve the sorting effect through a large flow rate, which may harm the erythrocytes and is more difficult to control, while the deformation of erythrocytes in chip B is more in line with expectations. At the same time, the contact between the erythrocytes and the pillar at a lower flow rate is more gentle, causing very low damage to the erythrocytes, which is more favorable to the sorting and enrichment of the cells.

Whether the enrichment efficiency in the experimental results can be further improved still needs to be further explored. In order to save on research costs and at the same time better analyze the details of the flow field in the chip, the flow simulation will be carried out by referring to the DLD array in the experiment.

## 4. Simulation of Erythrocyte Motility Based on the Finite Element Method

We simulated the flow in the experiment using the finite element method to better analyze the details of the erythrocyte interaction with the fluid and pillars in the chip. To save computational costs, we used 2D simulations to simplify the computational process, modeling the chip used in the experiments by creating an array of four rows with an in-chip channel height of 15 µm.

**Figure 12:**
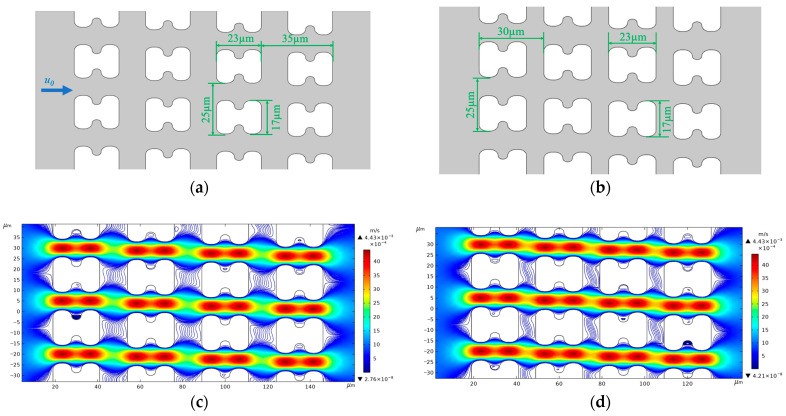
Structure size and streamline distribution of simulated DLD array. (**a**) Structure and dimensional details of the A-chip model, *u*_0_ is the fluid flow velocity at the inlet, and the flow direction is the same in all simulations; (**b**) Structure and dimensional details of the B-chip model; (**c**) Streamlines and flow velocity distributions in A-chip for an inlet mean flow velocity of 1 mm/s; (**d**) Streamlines and flow velocity distributions in B-chip for an inlet mean flow velocity of 1 mm/s.

We used version 5.6 of COMSOL^®^ Multiphysics software for flow simulation. To restore the flow in the experiment, we established two 2D array structures, as shown in Figure 12a,b, based on the actual top view shapes of chip arrays A and B, to serve as the simulation model of the chip internal structure. Figure 12c,d show the flow and streamline distributions of the two different I-pillars at an average inlet flow rate of *u*_0_ = 1 mm/s.

**Figure 13:**
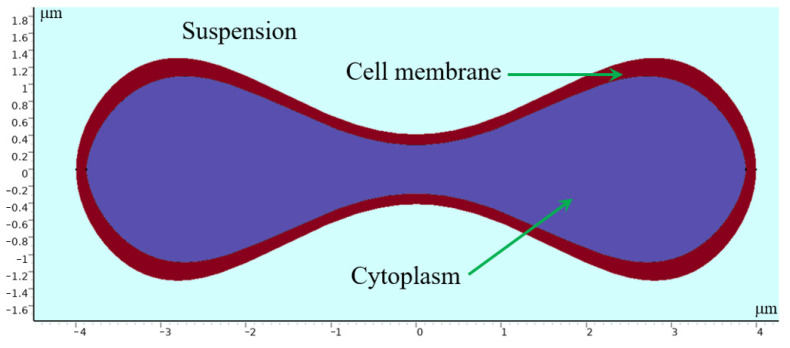
Geometry of 2D erythrocyte model.

Erythrocytes have a unique biconcave pie-shaped structure with a highly elastic cell membrane about 5 nanometers thick, consisting of a phospholipid bilayer with a protein backbone. The cytoplasm inside can be regarded as an incompressible viscous fluid [28,29,30]. In modeling erythrocytes, we use parametric curves to draw the shape of the erythrocyte cross section. Evans and Feng [31] derived approximate parameter curves from data on the shape of human erythrocytes:

(1) x=0.5Rsinω

(2) $$y=0.25R\cos\omega (c_{0}+c_{1}\sin^{2}\omega +c_{2}\sin^{4}\omega )$$

where 0≤ω≤π, $c_{0}=0.207$, $c_{1}=2.003$, $c_{2}=-1.123$, and R= 4 μm is the radius of the erythrocyte. By processing the parametric curves, an erythrocyte model with a similar shape of the erythrocyte was obtained, whose geometry is shown in Figure 13.

The thickness of the cell membrane (about 5 nm) is about 1 in 400 of the thickness of an erythrocyte, and to simplify the computational process, we modeled the erythrocyte as a structure consisting of a thicker linear-elastic membrane wrapped around an incompressible viscous fluid, and set the density of both parts to be 1090 kg/m^3^ [32], which is the same as that of the actual erythrocyte. The results obtained under the condition of *u*_0_ = 0.5 mm/s are in line with the expectation of sorting different deformable erythrocytes. Therefore, the flow simulation is designed based on this set of experiments.

**Figure 14:**
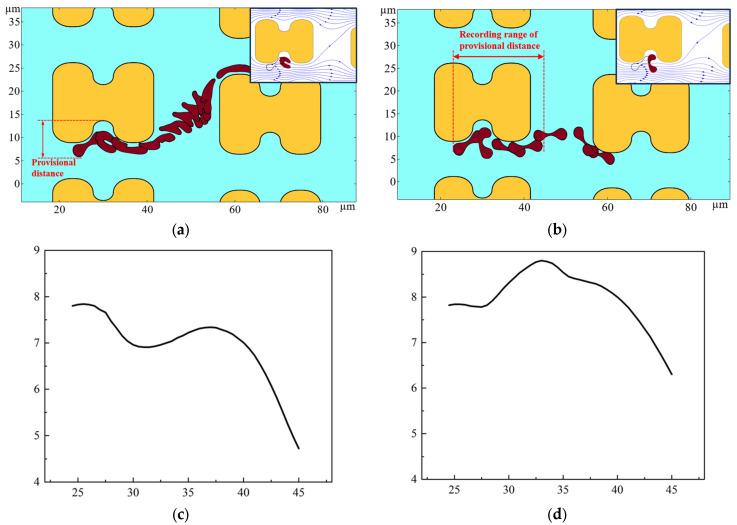
Motion of the two erythrocyte models during the flow simulation. (**a**) deformation and path of the softer erythrocyte model, the upper right corner also shows the position of the cell model in relation to the surrounding flow line when it is near the pillar; (**b**) deformation and path of the stiffer erythrocyte model, the upper right corner shows the position of the erythrocyte model in relation to the surrounding flow line when it is near the pillar; (**c**) provisional distance of softer cells; (**d**) provisional distance of harder cells.

The erythrocyte model was placed in a simulated flow channel, and after grid independence verification, the cell membrane thickness and Young’s modulus were varied to perform multiple sets of simulations. The deformation and path of the erythrocyte model in the simulation results were compared with the experimental results. Finally, a thickness of 0.05 and a Young’s modulus of 4000 Pa were chosen as the cell membrane parameters for softer cells; a thickness of 0.05 and a Young’s modulus of 2 × 10^6^ Pa were chosen for harder cells. The trajectories and deformations of the erythrocyte model under these parameters are similar to the experimental results when the positions of the erythrocyte model are derived according to a certain difference in time, as shown in Figure 14. As shown in Figure 14a, the distance of the depression of the pillar from the farthest point of the erythrocyte model is defined as the provisional distance. Figure 14c,d show the change of the provisional distances during part of the movement of the two cell models.

As shown in Figure 14, the deformation and path of the two cell models are very different. As can be seen in Figure 14c,d, the provisional distances of the softer cells continued to decrease when near the pillar. However, the provisional distances of the harder cells had a tendency to increase, and were always larger than those of the softer cells. Combined with the deformation process of the two cell models, when bypassing the pillar, the softer erythrocyte model could not maintain the cake structure due to higher deformation; it was more inclined to fit the pillar movement, its provisional distances were lower, and it was more easily able to enter the zigzag mode. Rigid red cell models, on the other hand, acquire larger provisional distances during rolling motion and are more easily able to flow in displacement mode.

According to the simulation results, under a certain flow rate and array layout, the erythrocyte model will obtain different t provisional distances due to the difference in deformability, which will further affect the movement trajectories of the two erythrocyte cell models in the array.

## 5. Conclusions

By performing flow experiments of erythrocyte samples with different deformability in two types of chips, we found that deformability greatly influences the deformation and motion paths of erythrocytes in the I-pillar DLD array under high deformability conditions. The flow rate of the suspension plays a crucial role in the sorting process of erythrocytes. Considering and leveraging the impact of deformability in the design of erythrocyte sorting chips can lead to the enrichment of diseased erythrocytes.

Meanwhile, in the flow simulation, we found that there is a large difference in the effect of the fluid on erythrocytes with different deformability when they tumble in the array. The interactions between the micropillar, the flow field, and the erythrocytes all affect the final flow trajectory of the erythrocytes. By continuously exploring the layout of the array in the chip and the effects caused by the flow rate of the suspension, the accuracy of the sorting can be continuously improved, which can provide a new, simple, and portable method for the diagnosis of blood disorders.

## Figures and Tables

**Table 1:** Deformation of erythrocytes in each group of experiments and the main paths of their movement.

Syringe Pump Injection Rate	Chip	Deformation and Path of 1D Erythrocytes	Deformation and Path of 7D Erythrocytes
4.5 μL/min	A	Parachute, displacement mode	String ball, zigzag mode
	B	Parachute, displacement mode	String ball, displacement mode
3 μL/min	A	String ball, zigzag mode	String ball, zigzag mode
	B	String ball, displacement mode	String ball, displacement mode
1.5 μL/min	A	Pie, zigzag mode	Pie, displacement mode
	B	Pie, zigzag mode	Pie, displacement mode

**Table 2:** Enrichment ratio of three kinds of erythrocytes in A and B microarray.

Erythrocyte Storage Time	Syringe Pump Injection Rate	Chip A Enrichment Ratio	Chip B Enrichment Ratio
1D erythrocytes	4.5 μL/min	12.14	15.29
	3 μL/min	5.26	17.55
	1.5 μL/min	7.27	6.91
7D erythrocytes	4.5 μL/min	3.89	16.17
	3 μL/min	9.33	17.31
	1.5 μL/min	14.82	19.24

## Data Availability

Data are contained within the article.
